# *Plasmodium vivax* Malaria and G6PD Testing

**DOI:** 10.3390/pathogens12121445

**Published:** 2023-12-13

**Authors:** Benedikt Ley, Lucio Luzzatto

**Affiliations:** 1 Global and Tropical Health Division, Menzies School of Health Research, Charles Darwin University, Darwin, NT 0811, Australia; 2 Department of Haematology, University of Firenze, 50134 Florence, Italy; 3 Department of Hematology and Blood Transfusion, Muhimbili University of Health and Allied Sciences, Dar es Salaam 65001, Tanzania

Early malaria investigators were certainly correct in classifying the species *falciparum* and the species *vivax* as belonging to the same genus, *Plasmodium*. Both are intra-erythrocytic in the human host through an important part of their life cycle, and they resemble each other a lot more than other species belonging to the same genus. Nevertheless, ever since *Plasmodium vivax (P. vivax)* and *P. falciparum* have been recognized under the microscope, over a century of studies have brought to light important biological and clinical differences, some of them rather spectacular; here, we will only mention three. First, *P. falciparum* parasitaemia can range from sub-microscopic to very high, whereby up to 50% of all red cells are parasitized; in contrast, *P. vivax* infects reticulocytes selectively, and it is rare to see more than 4% of all red cells parasitized. Second, any individual *P. falciparum* attack, if untreated, is potentially lethal in a non-immune person, whereas mortality from *P. vivax* is usually the consequence of recurrent attacks [1]. Third, during its development in the human host, *P. falciparum* has only one major choice to make: after entering the erythrocyte, it will opt for either the asexual or the sexual pathway. *P vivax*, on the other hand, must make an additional choice already at an earlier stage in the human host: once a sporozoite has entered a hepatocyte, the parasite has the option to complete schizogony (whereby merozoites will then invade red cells) or stay dormant and become a hypnozoite, that can reactivate weeks to months after the primary infection. From available evidence, we can infer that following infection, a significant fraction of parasites develop into hypnozoites, thus setting the stage for one or more relapses in the future.

This last point is very important not only with respect to clinical course and management but also with respect to pharmacogenetics. For the radical cure of *P. vivax*, it is necessary to administer, in conjunction with schizontocidal treatment, a drug that will effectively remove hypnozoites. The only drugs that can do this hitherto are the 8-aminoquinolines (8AQs) primaquine (PQ) [2] and tafenoquine (TQ) [3]. PQ has an elimination half-life of four to six hours and must be given over several days, whereas the elimination half-life of TQ is much longer at 12 to 13 days, and therefore, it can be given as a single dose [4,5,6]. While well tolerated in the majority of recipients, 8AQs can cause acute haemolytic anaemia (AHA) in persons who are G6PD deficient, including heterozygous females [7,8,9]. The prevalence of G6PD deficiency is high in most *P. vivax*-endemic areas [10,11,12].

In the face of this situation, different trends can be entertained. At one end of the spectrum, there is a view that the public health burden imposed by *P. vivax* malaria on the population as a whole is so great and eliminating malaria so urgent that there should be no impediment to the use of PQ or TQ, especially since the number of life-threatening drug-induced AHA cases will be small compared to the number of people protected from potentially life-threatening *P. vivax* relapses. At the other end of the spectrum, standards of good clinical practice—as well as WHO recommendations—demand G6PD testing before providing PQ or TQ [6] and/or daily supervision when administering either of these drugs; this requires significant resources in terms of health personnel and testing facilities. This Special Issue of *Pathogens* focuses on the important topic of how to reconcile effective and safe radical cure in areas where *P. vivax* is a major public health problem and G6PD deficiency is prevalent.

The paper by Pfeffer et al. assesses the quantitative expression of G6PD activity in genotypically confirmed hemizygotes and heterozygotes for 24 G6PD mutations, some common and some rare [13]. The authors make the important point that for each variant, the observed activity values span current classification thresholds. This article, together with that by Nannelli et al. [14], provided much of the evidence on which the World Health Organization based their recent revision of the classification of G6PD variants that combined the former class II and class III variants into one group.

Sadhewa et al. [15], with authors from Australia, Thailand, and the UK, review logistics and policies around G6PD testing. The authors illustrate that in different endemic areas, policies for persons with normal, deficient, and intermediate G6PD levels differ, and in some areas, policies are lacking.

Gerth-Guyette et al. [16], with authors from Vietnam and the USA, assess the feasibility and costs of integrating the diagnosis of G6PD deficiency through a point of care (POC) G6PD testing device by SD Biosensor (STANDARD G6PD; ROK) into routine care in Vietnam. The authors found that health care providers (HCP) can use this device in routine practice if continuous monitoring is ensured. The authors also found that costs per test increased with a decreasing malaria caseload. Adhikari et al., with authors from Thailand, the UK, and Australia, assessed whether diagnostic precision differed depending on the education level of the end user [17]. The authors report good agreement between the results obtained with the same SD Biosensor device by specially trained laboratory technicians and by malaria village workers in Cambodia. Brito-Sousa et al. [18], with authors from Brazil and the USA, report how HCPs, after appropriate training, were able to use this device in malaria treatment units in two municipalities of the Brazilian Amazon region. When the G6PD results were cross-checked by DNA analysis, no G6PD mutations were found in samples reported as normal by the SD Biosensor test, but, surprisingly, mutations were found in only 37% of the samples reported as G6PD deficient. Post-training proficiency of HCPs was better than 70% after a 4 hour training, and the test was well accepted.

The G6PD diagnostic by SD Biosensor is a major advance. To the best of our knowledge, it is the first device that returns G6PD activity normalized to haemoglobin concentration (Hb) at the point of care. The test is labelled as quantitative on the SD Biosensor website, but although it presents G6PD activity in IU/gHb, it is referred to as semi-quantitative on the PATH website, with cut-offs provided to define normal, intermediate, and deficient G6PD activities. According to the PATH website, “samples that generate a G6PD deficient or intermediate result should be assayed using a quantitative G6PD test to verify a deficiency” (https://www.path.org/programs/diagnostics/faq-standard-g6pd-test-sd-biosensor/: last accessed on 11 December 2023); this instruction, if followed, would defeat the POC concept. A limitation of the device from SD Biosensor is that results are not reliable if Hb readings are less than 7 g/dL; this precludes its use in patients with severe anaemia. Several papers in this Special Issue provide support for the clinical and public health value of this G6PD test, and fortunately, in each one of these papers, the authors declare no conflicts of interest. We think the trade name STANDARD G6PD is not appropriate because it could mislead people to gloss over the fact that the gold standard for measuring G6PD activity is the spectrophotometric assay [19]. In fact, the SD Biosensor analyser does not measure the characteristic absorption band of NADPH, the product of the G6PD reaction, at 340 nm. Rather, the measurement is based on the reduction of 5-bromo-4-chloro-3-indolyl-phosphate (BCIP) that is reduced to violet nitro blue tetrazolium (NBT) in the presence of the G6PD enzyme; the color intensity can be expected to be directly proportional to G6PD activity up to a point, but it is likely to reach saturation when G6PD activity is high [20].

Kosasih et al. [21], with authors from Indonesia, Australia, France, the Solomon Islands, Thailand, and the UK, report the consequences of having missed a diagnosis of G6PD deficiency. They report on five cases of PQ-induced acute haemolytic anaemia that occurred in G6PD deficient males enrolled in four clinical trials in Indonesia, the Solomon Islands, and Vietnam. These patients had been wrongly classified as G6PD normal, and in four of them, drug-induced haemolytic anemia was severe enough to require blood transfusion [21]. This paper illustrates that no diagnostic test is perfect; while all patients recovered without sequelae the outcome might have been different outside a well-conducted clinical trial.

Finally, Kartal et al. [22] compiled a large body of work on serological data accruing from epidemiologic studies of *P. vivax* malaria. The authors conclude that sero-surveillance is feasible and a powerful tool to monitor the progress of individual *P. vivax* elimination campaigns; however, much work remains to be done with respect to the standardization of methodologies.

There is an understandable tendency of regulatory bodies to try and dichotomize the use of a drug as ‘safe’ or ‘unsafe’. In the case of PQ and TQ, it would be nice to classify persons as having ‘risk of hemolysis’ or ‘no risk of hemolysis’. In reality, dealing with an X-linked gene is somewhat different. The phenotypic classification of persons as G6PD normal, intermediate, or deficient, as provided by G6PD testing, including by the SD Biosensor G6PD analyzer, is correct, but we must remain aware that the majority in the intermediate group are women heterozygous for G6PD deficiency [23] who have red cell mosaicism [24] (there will be a minority of males in the intermediate group, some who are simply at the lower end of the Gaussian distribution of G6PD activity of normal males and some who are G6PD deficient, but, for intercurrent reasons (e.g., haemolysis and reticulocytosis), have transiently increased G6PD activity). This means that a female in the intermediate group has, on average, about one-half G6PD normal red cells and about one-half G6PD deficient red cells; the latter are vulnerable to hemolysis when exposed to 8AQs. Therefore, these females will hemolyse when exposed to oxidant stressors, including PQ and TQ [8,25]; however, in the majority of cases, the degree of haemolysis will be less severe than in hemizygous G6PD deficient males or homozygous G6PD deficient females. This is reflected in the most recent WHO malaria treatment guidelines that recommend routine testing for G6PD deficiency prior to radical cure. Based on the test result, PQ is offered at 0.25–0.5 mg/kg body weight over 14 days to G6PD normal patients; the same dose, supervised, is provided to intermediate females, while patients diagnosed as G6PD deficient are treated over the course of eight weeks with a weekly dose of 0.75 mg/kg of body weight. Shorter courses of PQ at higher doses for G6PD-normal individuals are currently being assessed; the guidelines do not contain a recommendation on TQ [6], but the need for G6PD testing is in the TQ label. Thus, with respect to optimal patient management, it would seem reasonable that G6PD normal persons receive TQ, G6PD deficient persons do not receive TQ, and those in the intermediate group receive TQ only if medical supervision can be provided.

There is good evidence that *P. falciparum* has been an agent of Darwinian selection for G6PD deficient mutants [26,27,28,29]; the same has also been suggested with respect to *P. vivax* [30,31,32] when a decreased parasite density was found in subjects with the G6PD Mahidol variant compared to controls [30]. However, decreased parasite density is not, by itself, proof of decreased patient mortality. Bancone et al. have reported normal growth, development, and high parasite density of *P. vivax* in G6PD Mahidol reticulocytes in vitro [33]. G6PD activity in red blood cells declines over time, with the highest activity found in young reticulocytes [34]. Since *P. vivax* almost exclusively invades reticulocytes that have relatively high G6PD activity even in G6PD deficient individuals, the host G6PD status may be less relevant for the success of *P. vivax* infection compared to *P. falciparum*. While it cannot be ruled out that G6PD deficiency influences the clinical course of a *P. vivax* infection, the impressive geographic correlation between malaria endemicity and G6PD deficiency may mostly reflect the past selection through *P. falciparum*, even in areas where this parasite species is no longer prevalent [10].

While G6PD deficiency offers some form of protection against malaria, it carries with it the risk of haemolytic anaemia. The complex relationship between *P. vivax*, 8-aminoquinolines, and G6PD deficiency might be visualized metaphorically, as though they are at the three corners of a triangle (Figure 1). Malaria selects for *G6PD* mutant alleles that cause G6PD deficiency; PQ and TQ are effective in eliminating malaria hypnozoites but can cause acute haemolytic anaemia in G6PD deficient persons. The only licensed hypnozoitocides thus far are 8-aminoquinolines, and it is likely that they kill hypnozoites via the same oxidative damage that causes the haemolysis of G6PD-deficient red cells. An outstanding challenge is to dissociate these two effects: to identify a drug that kills hypnozoites but does not harm G6PD deficient red cells.

## Figures and Tables

**Figure 1:**
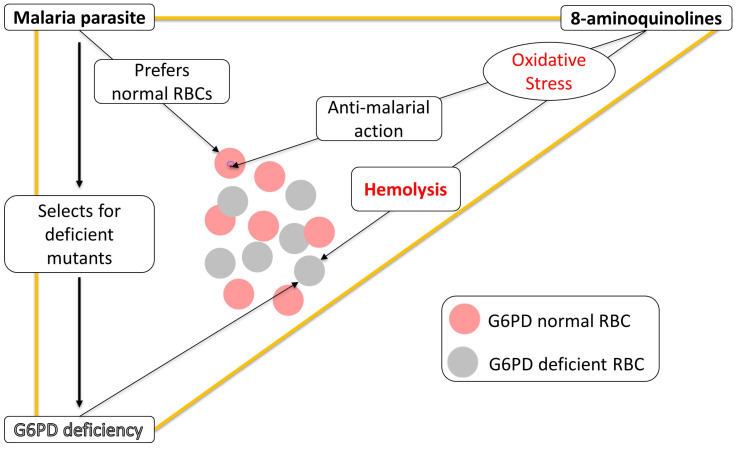
Genetics and pharmacogenetics of G6PD deficiency: a triangular relationship. One vertex of the virtual triangle is G6PD deficiency due to a mutation in the human G6PD gene located on the tip of the long arm of the X chromosome (Xq28). Like any other gene, G6PD is susceptible to spontaneous mutations, and some of these entail G6PD deficiency. Another vertex is the malaria parasite; when it infects heterozygotes, in whom G6PD normal red cells (pink) and G6PD deficient red cells (grey) co-exist, this mosaicism is key to protection [28], at least in the case of *P. falciparum*. As a result, genetically determined G6PD deficiency tends to become prevalent in malaria-endemic areas. The third vertex are 8-aminoquinolines, potent hypnozoitocides that, by producing reactive oxygen species (ROS), kill P. vivax hypnozoites [35]; unfortunately, ROS also cause oxidative injury to G6PD deficient red cells, thus causing acute haemolytic anaemia in G6PD-deficient persons. RBC = red blood cell.
